# Temperature Replica-Exchange Molecular Dynamics Reveals a Heterogeneous Recognition-Compatible Ensemble of the Laminin-Derived Peptide CDPGYIGSR

**DOI:** 10.3390/biom16070954

**Published:** 2026-06-27

**Authors:** Carmen Di Giovanni, Antonio Lavecchia

**Affiliations:** Department of Pharmacy, “Drug Discovery Laboratory”, University of Naples “Federico II”, 80131 Naples, Italy; cdigiova@unina.it

**Keywords:** nonapeptide, replica-exchange molecular dynamics, structural ensemble, 67 kDa laminin receptor, tumor cell invasion

## Abstract

The laminin-derived nonapeptide CDPGYIGSR contains the bioactive YIGSR motif, historically associated with inhibition of tumor cell adhesion, invasion, angiogenesis, and laminin-receptor-mediated cell responses. Although these activities have often been attributed to the 37/67 kDa laminin receptor/RPSA axis, the molecular identity and organization of the laminin-binding receptor system remain debated. This uncertainty makes it essential to define the intrinsic conformational preferences of CDPGYIGSR in solution before assigning a unique receptor-bound structure. In this study, temperature replica-exchange molecular dynamics (T-REMD) simulations in explicit solvent are employed to characterize the solution conformational ensemble of CDPGYIGSR. Free energy landscape analysis, clustering, and structural descriptors reveal a predominant compact bend-like backbone arrangement, together with alternative low-lying conformational states within a heterogeneous ensemble. Rather than assuming a single bioactive conformation, the conformational ensemble is analyzed in terms of structural features that are consistent with available NMR observations and reported structure–activity relationships. Importantly, the most populated conformations in solution do not necessarily correspond to the bioactive state upon receptor binding. Instead, a subset of conformations sharing common structural motifs, including a central backbone bend and specific residue exposure patterns, may represent states compatible with receptor recognition. These results provide an ensemble-based structural framework that connects simulation-derived conformational motifs with available NMR observations and structure–activity data, supporting a recognition-compatible ensemble model in which compact preorganized states may contribute to receptor binding.

## 1. Introduction

Laminins are extracellular heterotrimeric glycoproteins found in all basement membranes, specialized matrices associated with epithelia, endothelia, muscle cells, adipocytes, and peripheral nerves. They consist of different combinations of α, β, and γ chains and assemble into large molecules (400–900 kDa) with a characteristic cross- or T-shaped structure. To date, five α, four β, and three γ chains, along with splice variants, have been identified, generating at least 16 laminin isoforms in mammals (laminins 1–15) [1]. Because laminin nomenclature has subsequently been revised to indicate α, β, and γ chain composition, laminin-1 is now commonly referred to as laminin-111 [2]. Laminin-1 is a major component of basement membranes and contains several bioactive domains that interact with integrin and nonintegrin receptors, regulating cell adhesion, migration, proteinase secretion, and tumor metastasis [3,4].

One of its main receptors is the 67 kDa laminin receptor (67LR) [2], which is overexpressed in malignant cells compared to normal tissues [3,5,6,7]. Tumor cells also release a soluble form of 67LR in elevated amounts [8], and this increase correlates with highly invasive and metastatic phenotypes, making it a potential biomarker of tumor aggressiveness [2,3,5,8,9,10]. However, the molecular identity and structural organization of the receptor historically referred to as 37/67LR remain incompletely resolved. The 37 kDa laminin receptor precursor is now commonly associated with RPSA, whereas the origin, composition, and biochemical transition to higher-molecular-weight laminin-binding species, including 67LR, remain debated. This uncertainty suggests caution when assigning a unique receptor-bound peptide structure in the absence of explicit experimental binding restraints [11,12]. Although the interaction between laminin-1 and 67LR is crucial in cancer progression, its structural details remain unclear. For this reason, research has focused on short laminin-derived peptides as simpler models to study receptor binding and as potential anti-metastatic agents [13,14,15,16,17,18]. This strategy remains relevant because laminin-111 contains several bioactive sequences with distinct and sometimes opposing effects on tumor behavior, including YIGSR from the β1 chain, IKVAV and AG73 from the α1 chain, and C16 from the γ1 chain, each associated with distinct biological functions and receptor interactions [19,20]. Among these, the laminin-derived nonapeptide CDPGYIGSR-NH_2_ (hereafter referred to as peptide 11), derived from the β1 chain of laminin-1, has been proposed as a key binding sequence for 67LR [21,22]. Its C-terminal segment YIGSR has been shown to inhibit tumor cell invasion, reduce lung colonization, and suppress angiogenesis [23,24,25]. More recent work further supports the broader biological relevance of YIGSR-containing laminin-derived signals, including context- and concentration-dependent effects in cellular systems beyond tumor models [26]. These peptides act as disintegrins, blocking cell adhesion to the extracellular matrix [24]. Despite extensive studies, the relationship between the structure and activity of these peptides is not fully understood. A key unresolved question is whether functional recognition depends on the most populated solution conformations or on less populated states that become stabilized upon receptor engagement. This issue is particularly important for short flexible peptides, where biological activity may arise from an ensemble of recognition-competent conformations rather than from a single dominant solution structure. NMR experiments have reported different conformational preferences, highlighting variability in backbone organization [25,27,28], while additional studies indicate a tendency to form turn-like structures in solution [27]. These observations are consistent with the structure–activity analysis of peptide 11 analogs, which indicated that both side-chain presentation and backbone geometry contribute to anti-invasive activity, further complicating the identification of a single bioactive conformation [12]. Accordingly, CDPGYIGSR is expected to populate an ensemble of interconverting conformations in solution rather than a single dominant structure. However, a quantitative description of the full conformational ensemble in solution and its connection to experimentally observed structural heterogeneity remains incomplete, particularly in terms of linking ensemble populations to experimentally measurable observables. Importantly, the most stable structure in solution may not correspond to the biologically active, receptor-bound state. Therefore, analyzing the full conformational ensemble is essential to better understand their function. Molecular dynamics (MD) simulations are widely used to study biomolecular structure and dynamics, including the characterization of flexible bioactive peptides and peptide conformational ensembles [29,30]. Recent studies have highlighted the value of molecular modelling and enhanced-sampling approaches for understanding peptide structure, dynamics, and recognition mechanisms in systems where experimental structural information is limited [29]. Enhanced sampling methods such as temperature replica-exchange MD (T-REMD) improve conformational sampling by allowing simulations at multiple temperatures with exchanges between replicas [28,30]. This makes T-REMD particularly appropriate for CDPGYIGSR, where multiple low-energy conformational states, local turn formation, and rapid interconversion are expected to contribute to experimentally averaged observables.

Here, we use long-timescale T-REMD simulations in explicit solvent to explore the conformational ensemble of peptide 11. The specific aims are to identify recurrent structural motifs within the ensemble, evaluate their consistency with available NMR-derived observables, and assess whether these motifs can help rationalize available YIGSR-centered structure–activity data without assigning a single receptor-bound conformation. The analysis focuses on identifying dominant conformational features and their variability within the ensemble, rather than defining a single representative structure. It is important to note that, for flexible peptides, the most populated conformation in solution does not necessarily correspond to the bioactive conformation in the receptor-bound state. Instead, receptor binding may occur through conformational selection or induced fit mechanisms, involving one or more low-populated states within the conformational ensemble. Therefore, an ensemble-based description is essential to avoid overinterpretation of single-structure models.

## 2. Materials and Methods

### 2.1. Molecular Modeling and System Preparation

All molecular dynamics simulations were performed using GROMACS v4.5.3 [31] with the GROMOS96 force field. Although newer force fields optimized for intrinsically disordered peptides are currently available, GROMOS96 has been previously employed in simulations of short flexible peptides in aqueous solution, including REMD studies of conformational ensembles [32,33,34]. Therefore, the present study focuses on robust qualitative features of the conformational ensemble rather than absolute population distributions, which may depend on the force field choice [35,36]. Accordingly, emphasis is placed on reproducible structural motifs and ensemble-averaged properties rather than precise free-energy ranking of individual conformations. Initial peptide coordinates for peptide 11 were generated following the protocol reported by Nelson et al. [37]. Briefly, the structure of the YIGSR-binding peptide mEGF-(residues 33–41) (PDB ID: 1EPI) was used as an initial template. Residues outside the nonapeptide region were removed, and the remaining sequence was modified by in silico residue substitutions to obtain the laminin-derived CDPGYIGSR sequence. The resulting structure was subsequently processed, protonated, energy-minimized, and equilibrated prior to REMD simulations. Consistent with the experimental peptide sequence CDPGYIGSR-NH_2_, the C-terminus was modeled as an amide. The N-terminus was modeled as a neutral free amine rather than as an acetyl-capped terminus. Thus, no additional peptide-bond analog terminal caps were introduced, and the overall system was neutral without counter-ions. No explicit salt was added, as the small size of the peptide and the absence of a net charge minimize long-range electrostatic artifacts under periodic boundary conditions. Ionizable side chains were assigned standard protonation states, with Asp2 negatively charged and Arg9 positively charged. No alternative protonation states were considered because the dominant protonation states of Asp2 and Arg9 are expected to prevail under near-neutral conditions and were not anticipated to qualitatively alter the conformational ensemble. Energy minimization was performed using 1000 steps of the steepest descent algorithm to remove steric clashes. The peptide was solvated in a rectangular box of SPC water molecules [38] with a minimum solute–box distance of 1.4 nm. This distance was selected to exceed the 1.2 nm real-space cutoff employed for electrostatic and van der Waals interactions and to minimize the possibility of direct short-range interactions between the peptide and its periodic images. Periodic boundary conditions were applied in all directions to avoid finite-size effects. The simulation setup was consistent with the minimum-image convention used in GROMACS, according to which the non-bonded cutoff must remain smaller than half of the shortest box vector. Simulations were performed on hybrid CPU-GPU HPC systems at the CINECA supercomputing center.

### 2.2. Equilibration Protocol

Following minimization, the system was equilibrated through a restrained minimization and short MD run with harmonic restraints on solute atoms to allow solvent relaxation. Restraints were gradually released during equilibration to ensure a smooth transition toward unbiased dynamics. This was followed by stepwise heating using four short unrestrained MD simulations (50 ps each) at 50, 100, 200, and 250 K, progressively reaching the temperature range required for REMD (273–373 K).

### 2.3. Replica Exchange Molecular Dynamics (REMD)

T-REMD simulations [24,30,39] were performed in the NVT ensemble using 16 replicas distributed over the temperature range 273–360 K, chosen to ensure sufficient overlap of energy distributions between neighboring replicas and efficient exchange probabilities across the temperature ladder. In this approach, multiple replicas of the system are simulated at different temperatures and exchanged periodically according to a Metropolis criterion [40]. All structural analyses, conformational populations, clustering procedures, and free-energy projections discussed throughout this work were derived from the 300 K ensemble. Initial velocities were assigned from a Maxwell–Boltzmann distribution at the target temperature. Temperature control was achieved using the velocity-rescaling thermostat [41]. A coupling time constant of 0.1 ps was used for temperature control. Long-range electrostatics were treated using the particle mesh Ewald method with a real-space cutoff of 1.2 nm, and a Fourier grid spacing of approximately 0.12 nm with fourth-order interpolation. Bond constraints were enforced using the LINCS algorithm, and a 2 fs integration time step was used. Temperature sets were generated using a REMD temperature predictor [42], targeting an exchange probability of ~20%. The resulting temperature ladder consisted of 16 replicas at 273, 278, 283, 288, 294, 299, 305, 311, 317, 322, 329, 335, 341, 347, 353, and 360 K. The ladder was selected to maximize overlap between neighboring energy distributions and to achieve efficient replica exchange, rather than to sample only temperatures close to physiological conditions [28,38,41]. Exchange attempts between neighboring replicas were performed every 2 ps using the Metropolis criterion. Each replica was simulated for 70 ns, resulting in a total aggregated sampling time of 1.12 μs. Coordinates and energies were recorded every 2 ps for subsequent analysis. The average exchange acceptance probability between neighboring replicas was approximately 25–35%, indicating efficient replica mixing and conformational sampling. The use of multiple replicas exchanging across a temperature ladder provides repeated visits of the system to the 300 K ensemble from distinct thermal histories, improving conformational exploration relative to a single-temperature trajectory. REMD is particularly suited for systems with rugged free energy landscapes and multiple metastable states separated by high barriers. Convergence was assessed by monitoring structural observables, including RMSD, cluster populations, and principal components, over simulation time. Analyses were performed on equilibrated trajectory segments.

### 2.4. Trajectory Analysis

Principal component analysis (PCA) was performed on the Cartesian positional covariance matrix of Cα atoms after least-squares alignment to obtain a low-dimensional projection of the sampled conformational space [43,44], capturing the largest-amplitude conformational changes within the peptide ensemble. Free energy surfaces (FES) were constructed by projecting the trajectory onto the first two principal components. PCA was performed using *g_covar* and *g_anaeig* from GROMACS. The PCA was computed from 3353 trajectory frames extracted from the equilibrated portion of the 300 K trajectory (5–72 ns). Conformational clustering was carried out using the GROMOS algorithm implemented in the GROMACS *g_cluster* tool, based on pairwise backbone RMSD after structural alignment. A cutoff of 1.4 Å was used to identify distinct structural states. The GROMOS algorithm defines clusters by assigning structures within the selected RMSD cutoff to the conformation with the largest number of neighbors, iteratively removing assigned structures until all frames are classified. The cutoff was selected to provide a balance between structural resolution and meaningful cluster populations for this short flexible peptide. The same equilibrated 300 K trajectory segment was used for clustering and structural analyses, with coordinates sampled every 2 ps. Secondary structure content and solvent-accessible surface area (SASA) were calculated using DSSP [45], which assigns secondary structure based on hydrogen-bonding patterns and backbone geometry. Additional structural descriptors, including radius of gyration (Rg), intramolecular hydrogen bonds, and SASA, were used to characterize peptide compactness and stability. Backbone dihedral-angle distributions were analyzed by pooling the ϕ and ψ angles of residues 2–8, and the corresponding PMF was derived from the probability distribution of the sampled dihedral states. In the Ramachandran PMF map, neighboring contour lines were separated by 2 kJ mol^−1^. Hydrogen bond analysis was performed using a Perl script (plot_hbmap.pl). Free energy surfaces were generated using GnuPlot [46]. Plots were produced using GROMACS utilities and visualized with xmgrace [47]. Molecular visualization was performed using VMD [48] and PyMOL [49].

### 2.5. Comparison with Experimental Data

Ensemble-averaged interproton distances were calculated over the full simulation trajectories, weighted by conformational populations. Because NOE intensities reflect time- and ensemble-averaged distances, this approach allows a direct comparison between simulated and experimental observables. These values were compared with NOE-derived distance constraints from experimental NMR data to assess agreement between simulation and experiment. Deviations were interpreted in the context of peptide flexibility and the ensemble-averaged nature of NOE observables.

## 3. Results

### 3.1. Conformational Sampling and Secondary Structure Analysis

**Figure 1:**
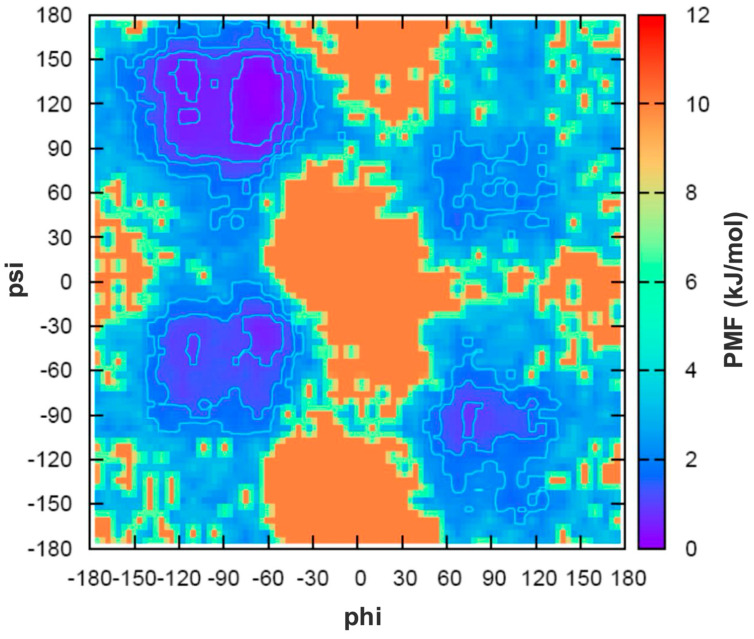
Potential of mean force (PMF) derived from the distribution of backbone dihedral angles (ϕ, ψ) for residues 2–8 of peptide 11 during the equilibrated portion of the T-REMD simulation. The PMF map describes the conformational free-energy landscape as a function of ϕ and ψ, with neighboring contour lines separated by 2 kJ mol^−1^. Low-free-energy regions correspond to the most highly populated backbone geometries sampled during the simulation. Because the map was generated from pooled φ/ψ values, it provides a global view of the sampled backbone conformational space rather than residue-specific Ramachandran distributions.

An unconstrained T-REMD simulation in explicit solvent was carried out starting from the modeled peptide structure described in the Methods section. Sixteen replicas were simulated for 70 ns each, yielding an aggregated simulation time of 1.12 μs. Efficient conformational sampling was supported by effective replica exchanges and stable exploration of the conformational space during the simulations. Consistent replica diffusion across the temperature ladder and the stability of major structural observables over the equilibrated trajectory supported the use of the 300 K ensemble for subsequent structural analysis. The 300 K ensemble is composed of trajectory segments originating from different replicas following exchange events, providing repeated visits to the target temperature from distinct thermal histories and supporting conformational exploration of the peptide ensemble. The conformational space sampled during the simulation was first analyzed through backbone dihedral angle distributions. A Ramachandran map was generated by pooling the ϕ and ψ angles of residues 2–8 over the equilibrated portion of the trajectory. The α-like, bridge, and β-like regions were assigned using conventional backbone dihedral definitions for peptide conformational analysis, with the β-like region corresponding to extended conformations in the upper-left region of Ramachandran space [50]. The corresponding potential of mean force is shown in Figure 1, with neighboring contour lines separated by 2 kJ mol^−1^. Because the Ramachandran PMF was generated by pooling residues 2–8, it should be interpreted as a global description of the backbone conformational space sampled by the central peptide region rather than as a residue-specific Ramachandran analysis. This distinction is relevant because the analyzed segment contains two glycine residues and one proline residue, whose backbone conformational preferences differ from those of standard amino acids [51,52]. Glycine has a broader accessible φ/ψ space because of its minimal side chain, whereas proline is conformationally restricted by its cyclic backbone. Therefore, the pooled map was used to identify the dominant backbone regions sampled by the peptide, whereas residue-specific structural tendencies were assessed through DSSP and RMSF analyses.

**Figure 2:**
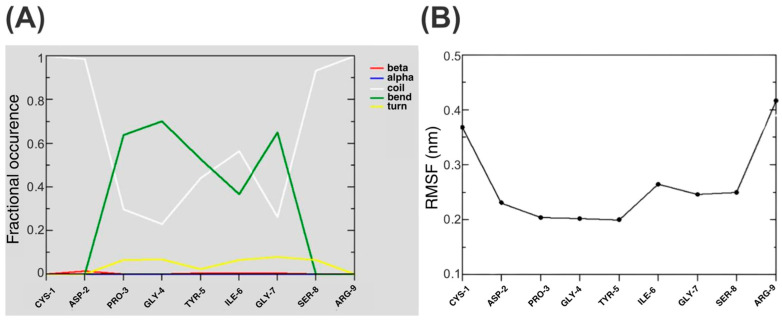
Secondary structure occurrence and flexibility of peptide 11. (**A**) Residue-wise fractional occurrence of DSSP-assigned secondary-structure states over the equilibrated T-REMD trajectory at 300 K. Values are reported on a 0–1 scale. (**B**) Root mean square fluctuations (RMSF) of Cα atoms relative to their average positions, highlighting differences in flexibility along the peptide sequence.

The analysis revealed that the peptide predominantly samples extended β-like regions of Ramachandran space, accounting for approximately 73% of the analyzed backbone conformations, while a smaller fraction, approximately 22%, falls within α-like regions. Despite the presence of α-like local backbone geometries, no significant helical content was observed. The predominance of β-like backbone geometries is consistent with the absence of persistent α-helical structure and with the tendency of the central peptide segment to adopt locally folded conformations, as further evidenced by the secondary-structure analysis presented below. Secondary structure analysis based on the DSSP algorithm [45] confirmed that peptide 11 does not exhibit stable α-helical conformations under the simulated conditions, indicating that the Ramachandran populations reflect transient local geometries rather than persistent regular secondary structure. Instead, the peptide is mainly populated by bend and coil structures, with bend conformations being the most prevalent structural element. The residue-wise secondary-structure occurrence profile (Figure 2A) indicates that bend conformations are concentrated in the central region of the peptide (P3–G7), whereas the terminal residues remain predominantly disordered. In particular, bend conformations are most frequently observed in the G4–G7 segment, with five out of nine residues assigned to bend states for more than 50% of the analyzed trajectory and peak bend propensities observed for G4 and Y5. The high bend propensity of G4 is relevant because previous structure–activity studies reported that substitutions at this position affect the biological activity of peptide 11 [15,53]. The location of Pro3 at the N-terminal side of this bend-prone region may further contribute to the observed conformational behavior. Because of its cyclic backbone, proline restricts the accessible φ angle and is frequently enriched in local chain reversals and turn/bend motifs, whereas glycine provides high backbone flexibility [52]. Accordingly, the P3–G4 region may act as a local conformational hinge that separates the flexible N-terminal portion from the more conformationally restricted G4–G7 segment. β-turn assignments are only weakly populated, indicating that the dominant structural tendency is better described as a dynamic bend rather than a persistent canonical β-turn.

The flexibility profile of the peptide was further characterized by calculating the root mean square fluctuations (RMSF) of Cα atoms (Figure 2B). The central segment (D2–S8), which contains the YIGSR motif, exhibits reduced fluctuations compared to the terminal residues, suggesting partial conformational restriction of the bioactive region within an otherwise flexible peptide.

### 3.2. Conformational Clustering and Free Energy Landscape

**Figure 3:**
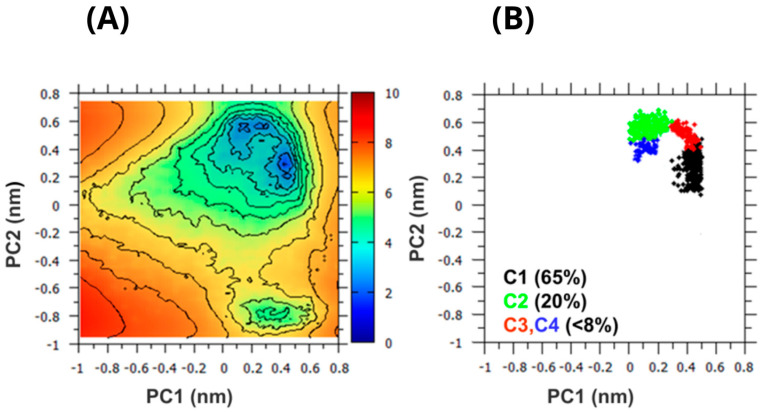
Free energy landscape and conformational clustering of peptide 11 based on Cartesian Cα PCA. (**A**) Free energy surface projected onto the first two principal components (PC1 and PC2) at 300 K. (**B**) Projection of the four most populated conformational clusters onto the PC1–PC2 space. Cluster populations are reported as percentages of the total ensemble, with Cluster 1 representing the most populated conformational basin.

To characterize the conformational ensemble in more detail, clustering analysis was performed based on backbone RMSD. Conformations differing by more than 1.4 Å were considered structurally distinct. This cutoff was selected to resolve the major conformational basins while avoiding over-fragmentation of a short and highly flexible peptide ensemble. The conformational landscape was further analyzed by projecting the trajectory onto the first two principal components (PC1 and PC2) [43,44] and constructing the corresponding free energy surface at 300 K (Figure 3A). The first two principal components accounted for approximately 57.1% of the total Cα positional variance and were therefore used to visualize the dominant conformational basins of the ensemble. The projections of the four most populated clusters onto the PC space are shown in Figure 3B.

The analysis reveals the presence of multiple low-energy minima in the conformational space, consistent with a heterogeneous ensemble rather than a single dominant solution structure. In the analyzed 300 K ensemble, Cluster 1 accounts for 58.6% of sampled conformations, Cluster 2 for 22.5%, Cluster 3 for 10.9%, and Cluster 4 for 5.9%, providing a quantitative estimate of the relative populations of the dominant conformational basins. In addition to the main basin populated by Clusters 1 and 2, the FES also shows a distinct low-energy region around PC1 ≈ 0.4 nm and PC2 ≈ −0.8 nm, corresponding to one of the lower-population conformational states identified by clustering. The free-energy surface therefore indicates the coexistence of multiple thermally accessible conformational states separated by relatively low free-energy barriers, suggesting that interconversion among these states is thermodynamically plausible within the sampled ensemble. Accordingly, the free-energy landscape should be interpreted as a population map of accessible conformational states rather than as a direct kinetic description of interconversion pathways.

### 3.3. Structural Features of the Dominant Conformational Cluster

**Figure 4:**
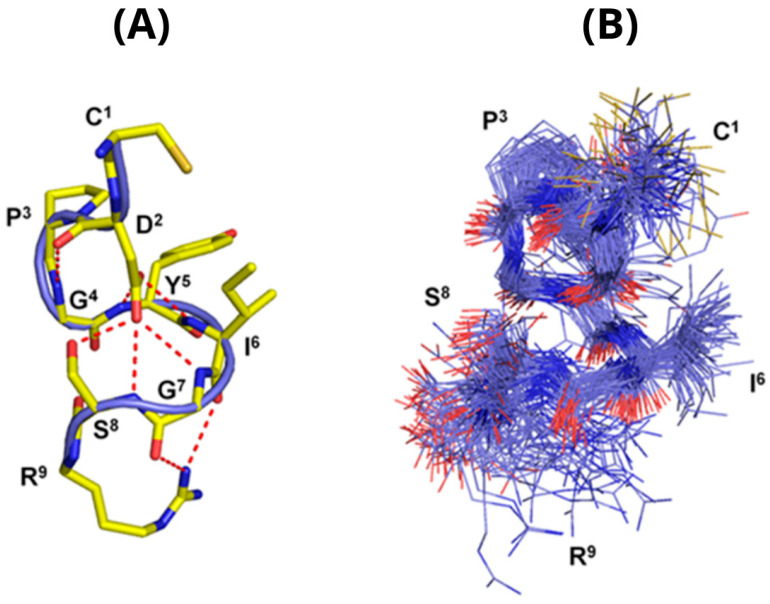
Structural features of the most populated conformational cluster (Cluster 1). (**A**) Representative centroid structure of Cluster 1. (**B**) Superposition of representative conformations belonging to Cluster 1. Despite local conformational variability, the central bend involving residues G4–S8 is conserved across the dominant ensemble.

The representative structure (centroid) of the most populated cluster (Cluster 1) is shown in Figure 4A, while Figure 4B illustrates the structural variability within this cluster. The superposition reveals that, despite local structural variability, the central bend involving residues G4–S8 is preserved across the dominant conformational ensemble.

Cluster 1 is characterized by a compact conformation with a bend centered on residues 4–8 (GYIGS), consistent with the NMR-derived proposal that peptide 11 can adopt bent conformations involving the YIGSR region [53]. This structural feature is also compatible with structure–activity data showing that backbone geometry around Gly7 influences anti-invasive activity [15]. In particular, experimental replacement of Gly7 by D-Ala preserved invasion-inhibitory activity, whereas Gly7 replacement by L-Ala markedly reduced activity, supporting the functional relevance of a bend-permissive local geometry in the YIGSR region [53]. Consistently, cyclic CDPGYIGSRC-NH_2_ analogs were reported to retain anti-invasive and anti-metastatic activity, and NMR modeling suggested that conformational restriction can favor an S-shaped backbone arrangement compatible with activity [15]. This conformational state is associated with a network of intramolecular hydrogen bonds. In particular, the side chain of residue D2 frequently forms hydrogen bonds with backbone amide groups of neighboring residues, including G4, Y5, I6, G7, and S8, with varying probabilities. The most recurrent contacts involve D2 with Y5 NH, G4 NH, I6 NH, G7 NH, and S8 NH, with occupancies of approximately 39%, 32%, 31%, 25%, and 15%, respectively. Additional D2-mediated contacts involving Y5, R9, and the D2(C=O)–G4(NH) backbone hydrogen bond are also observed, although with lower occupancies. The most recurrent intramolecular hydrogen bonds observed across the entire 300 K ensemble are summarized in Table 1. Although these values are ensemble averages rather than Cluster 1-specific quantities, they are dominated by interactions associated with the most populated compact conformational basin and therefore provide a useful description of the interaction network stabilizing Cluster 1-like conformations. Although individually transient, these interactions collectively define the dominant hydrogen-bond network associated with the compact bend-containing conformations of Cluster 1 and provide a structural basis for the persistence of the central G4–S8 bend motif. The recurrence of these interactions within the sampled ensemble supports their structural relevance despite their transient nature.

### 3.4. Secondary Conformational States and Salt Bridge Formation

**Figure 5:**
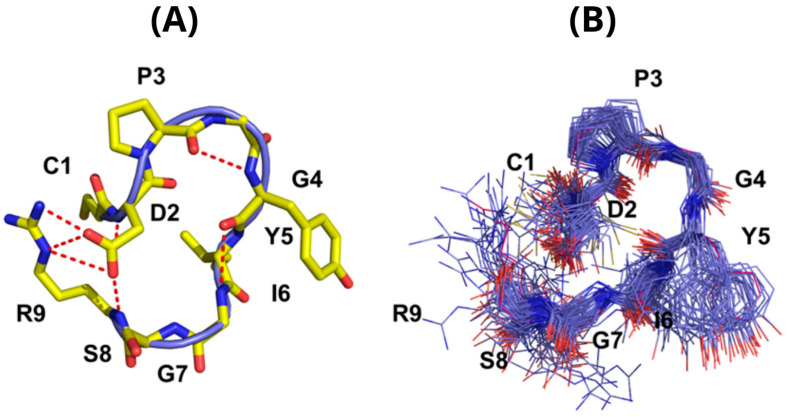
Structural features of the second most populated conformational cluster (Cluster 2). (**A**) Representative centroid structure of Cluster 2. (**B**) Superposition of representative conformations belonging to Cluster 2. The ensemble retains the central bend motif but displays increased variability in the relative orientation of the terminal segments and in the D2–R9 electrostatic interaction region.

The second most populated conformational cluster (Cluster 2) is shown in Figure 5. Similar to Cluster 1, it exhibits a structured central region and flexible termini. The conformational superposition shown in Figure 5B indicates that the central bend motif remains conserved within Cluster 2, whereas greater variability is observed in the terminal regions and in the relative orientation of residues involved in transient electrostatic interactions.

**Figure 6:**
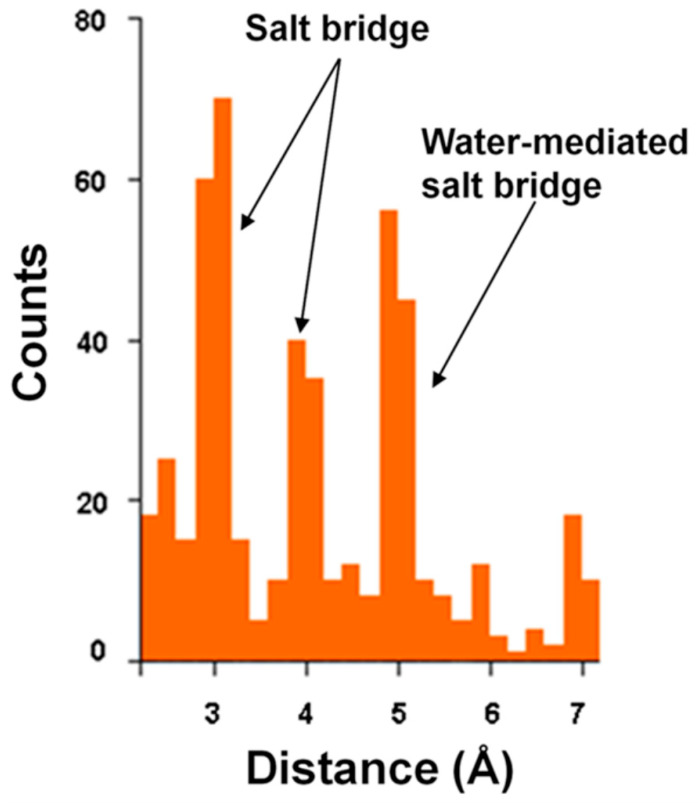
Distribution of D2–R9 charged-group distances observed in the 300 K ensemble. The histogram illustrates the coexistence of direct ion-pair contacts, solvent-mediated interactions, and more extended conformations, providing a structural signature of the secondary conformational basin represented by Cluster 2. The distribution was calculated over the entire 300 K ensemble rather than from Cluster 2 alone. The data indicate the presence of both direct salt bridges and solvent-mediated interactions within the conformational ensemble.

Cluster 2 also displays a bend involving residues 4–8, but differs in the detailed arrangement of intramolecular interactions. In this cluster, a transient electrostatic contact between residues D2 and R9 is frequently observed. The distribution of D2–R9 charged-group distances calculated over the entire 300 K ensemble is shown in Figure 6. Although discussed here in the context of Cluster 2, this distribution reflects the coexistence of all conformational states sampled during the simulation and therefore provides an ensemble-level description of the D2–R9 interaction.

Three main populations are observed, corresponding to direct ion-pair contacts (~3 Å), solvent-mediated interactions (~5 Å), and more extended conformations centered around ~7 Å. The latter population indicates that D2–R9 is not maintained as a persistent salt bridge across the ensemble but instead behaves as a transient electrostatic contact sampled by a subset of conformations. This classification is consistent with previous analyses showing that ion-pair strength depends strongly on charged-group geometry and that longer-range ion pairs are substantially weaker than direct salt bridges [54]. Accordingly, D2–R9 is best interpreted as a population-modulating electrostatic interaction rather than as a persistent structural constraint. Together, Table 1 and Figure 6 indicate that the two dominant conformational basins are stabilized by distinct interaction patterns, namely a distributed hydrogen-bond network in Cluster 1 and transient long-range electrostatic contacts in Cluster 2.

### 3.5. Role of Hydrophobic Interactions

**Figure 7:**
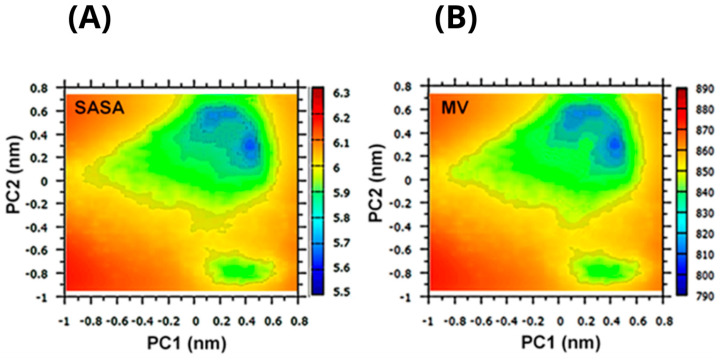
Hydrophobic contributions to conformational stability. (**A**) Solvent-accessible surface area (SASA, nm^2^) and (**B**) mean molecular volume (MV, Å^3^) projected onto the first two principal components (PC1 and PC2). Regions characterized by low SASA and molecular volume correspond to more compact conformations associated with low free energy basins.

To evaluate the contribution of hydrophobic effects to conformational stability, the mean molecular volume (MV) and solvent-accessible surface area (SASA) were mapped onto the PC1–PC2 space (Figure 7). Hydrophobic interactions are mediated by the solvent, and their magnitude and functional dependence are influenced by the size and shape of the solvated peptide. For solutes with diameters of approximately 20 Å or larger, these interactions scale with the solvent-accessible surface area, whereas for smaller solutes they are more closely related to the molecular volume [55,56].

Areas with reduced SASA and molecular volume values correspond to compact conformational states and coincide with the primary free energy minima identified in Figure 3. Notably, the most populated cluster (Cluster 1) exhibits reduced MV and SASA values, consistent with a relatively compact structural arrangement. Overall, these results suggest that hydrophobic effects are associated with compact conformational states within the ensemble. Given the small size of the peptide, this contribution should be interpreted as local solvent-exposure modulation rather than formation of a protein-like hydrophobic core.

### 3.6. Comparison with NMR Data

One of the few experimental structural investigations available for peptide 11 was reported by Ostheimer et al. in 1992 [53]. NOESY experiments performed in H_2_O/D_2_O revealed NOE cross-peaks between sequential backbone protons (αH_i_–NH_i+1_ and NH_i_–NH_i+1_). The protons within the CDPG sequence resonated at chemical shifts close to random coil values, indicating that this region is highly flexible in solution. In contrast, the YIGSR segment appeared less flexible than a random coil, suggesting partial conformational restriction for a significant fraction of the time in aqueous solution. Furthermore, peptide 11 was proposed to exhibit a tendency to adopt bent conformations around Gly7 within the YIGSR motif, which may be relevant for laminin binding to its metastasis-associated receptor [53]. The present simulations support and extend this interpretation: the bend is not observed as a single fixed structure, but emerges as a recurrent conformational motif within the dominant low-energy ensemble, particularly across the G4–S8 region. Thus, the T-REMD results support and extend the structural interpretation proposed by Ostheimer et al. by placing the inferred bend within a broader ensemble-based description of peptide flexibility. Subsequent conformational studies of laminin-1-derived peptides further emphasized that CDPGYIGSR and YIGSR display solvent-dependent conformational preferences and that conservative sequence changes, such as the C-terminal Arg-to-Lys substitution in YIGSR, can strongly affect antimetastatic activity, consistent with a recognition mechanism sensitive to both side-chain presentation and backbone organization [27]. Additional receptor-binding studies using the YIGSR-related mEGF-(33–42) peptide further suggested that spatial presentation of tyrosyl and arginyl residues may be relevant for recognition by YIGSR-specific laminin-binding receptor systems [37].

To enable a meaningful comparison with experimental data, interproton distances were calculated as ensemble averages over the conformational ensemble, weighted according to their respective populations. This approach reflects the ensemble-averaged nature of NOE measurements, which report on population-weighted structural features rather than individual conformations. The experimental NMR measurements were acquired under mildly acidic conditions, whereas the simulations employed fixed protonation states. However, Asp2 is expected to remain predominantly deprotonated across this pH range because its model pKa is close to 4.0, while the peptide C-terminus is amidated (CDPGYIGSR-NH_2_) and therefore not titratable. Consequently, the comparison remains informative for assessing the overall agreement between ensemble-averaged structural features and NOE-derived observables. This interpretation is consistent with current views of solution NMR data for flexible molecules, where time-averaged observables are best interpreted through conformational ensembles rather than single structures [57]. The comparison between experimental and simulated distances is presented in Table 2. Because multiple conformational basins contribute to the sampled ensemble, some interproton distances are expected to display broad or multimodal distributions. Consequently, the values reported in Table 2 should be interpreted as ensemble-averaged descriptors rather than distances associated with a single dominant conformational state.

The mean absolute deviation across the listed contacts is approximately 0.40 Å. Three contacts show deviations larger than 0.7 Å, indicating local discrepancies in the Y5–S8 region, whereas the remaining contacts are reproduced within approximately 0.6 Å.

Overall, the simulated distances reproduce the general pattern of short-range contacts observed experimentally, although the agreement is not uniform across all proton pairs. A small number of larger discrepancies are observed, mainly involving local contacts within the Y5–S8 region, which may be attributed to the intrinsic flexibility of the peptide as well as to the ensemble-averaged character of NMR measurements. Taken together, these results indicate that the simulated conformational ensemble reproduces several ensemble-averaged features observed experimentally, even though no single conformation fully satisfies all the NOE constraints. This supports the interpretation that peptide 11 should be described as an ensemble of interconverting conformations rather than by one dominant solution structure, with the YIGSR-containing segment sampling partially preorganized states that are consistent with available NMR and structure–activity data. Thus, the main contribution of the present analysis is not the identification of a unique bioactive structure, but the integration of conformational sampling, NMR-derived observables, and structure–activity evidence into a coherent ensemble-based interpretation of peptide 11.

## 4. Discussion

The present results indicate that peptide 11 does not adopt a single well-defined structure in aqueous solution, but rather exists as a dynamic ensemble of interconverting conformations [28,29]. Within this ensemble, multiple low-energy states are populated, with a dominant conformational basin characterized by a compact backbone bend involving residues 4–8. The proximity of this bend to Pro3 and its inclusion of G4 are noteworthy because proline and glycine residues frequently contribute to local chain reversals and turn/bend motifs [52]. This interpretation reinforces the structure–activity relevance of the G4–YIGSR region, since substitutions at G4 and Gly7 have been reported to modulate anti-invasive activity [15,53]. This finding is consistent with the NMR-derived proposal by Ostheimer et al. that peptide 11 samples bent conformations in the YIGSR region and with the observation that analogs preserving a bend-permissive geometry around Gly7 retain anti-invasive activity [53]. Importantly, the present simulations emphasize this bend as a recurrent ensemble feature rather than as a single static solution conformation. Accordingly, the main contribution of the present analysis is not the identification of an entirely new bend, but the demonstration that the experimentally inferred bend is embedded within a heterogeneous conformational ensemble containing both dominant and secondary compact states. Enhanced-sampling simulations provide a framework for reinterpreting historical peptide structure–activity data in terms of conformational ensembles rather than single representative structures. While the existence of bend-like conformations was already suggested by NMR studies, the relative populations of alternative low-energy states and their associated interaction networks were not previously characterized. The recurrent bend motif is consistently observed across clustering, free-energy, and secondary-structure analyses, suggesting that it represents a recurrent feature of the peptide’s conformational behavior. The population of these compact states is likely favored by a combination of transient intramolecular hydrogen bonds, local solvent-exposure changes, and backbone flexibility, rather than by a single persistent stabilizing interaction. At the same time, the presence of several thermally accessible minima separated by relatively low free energy barriers indicates significant conformational plasticity. This flexibility is particularly relevant for interpreting the functional role of the YIGSR motif, which has historically been associated with laminin-receptor-mediated cell attachment and anti-metastatic responses [58,59,60,61]. However, the receptor system commonly referred to as 37/67LR or RPSA remains structurally and biochemically complex, with persistent ambiguity regarding the relationship between RPSA, laminin-binding activity, and higher-molecular-weight receptor species. This uncertainty further supports a peptide-centered interpretation of the present simulations rather than direct assignment of receptor-bound states [11,12]. Comparison with available NMR data [53] shows that the simulated ensemble captures the general pattern of short-range NOE-derived contacts, while local deviations in the Y5–S8 region indicate that no single conformer can account for all experimental observables [62]. While some deviations are observed, they remain within a range expected for highly flexible peptides and likely reflect both force field limitations and the intrinsic time-averaged nature of NMR observables. Importantly, no single conformation satisfies all experimental restraints, further supporting an ensemble-based description of peptide 11 in solution. This interpretation is also consistent with subsequent solution NMR studies of laminin-1-derived peptides, which emphasized solvent-dependent conformational preferences for CDPGYIGSR and YIGSR rather than a single invariant structure. In particular, these studies support the idea that the bioactive YIGSR segment is conformationally plastic but can sample preferred local geometries that are sensitive to sequence context, solvent environment, and conservative substitutions such as Arg-to-Lys at the C-terminus [23]. To the best of our knowledge, no more recent high-resolution experimental structure of CDPGYIGSR in aqueous solution has superseded the available NMR studies of peptide 11 and related YIGSR-containing analogs. Thus, the present work provides an updated computational reinterpretation of the existing NMR and structure–activity evidence using an ensemble-based framework.

The present ensemble-based results also extend earlier computational studies on laminin-derived peptides. Previous molecular dynamics simulations of CDPGYIGSR and related YIGSR-containing sequences suggested that local folding, solvent exposure, and side-chain presentation contribute to the structural behavior of this motif. The present T-REMD analysis improves on these earlier approaches by explicitly sampling multiple low-energy basins and by comparing the resulting ensemble with available NMR-derived observables [21,63]. The recurrent compact bend observed here is also consistent with prior SAR-driven design work showing that conformationally constrained cyclic analogs of peptide 11 can retain biological activity, supporting the idea that activity depends on accessible backbone geometries rather than on a completely disordered peptide chain [15]. Importantly, the present analysis indicates that this bend is not associated with a single dominant conformation but is instead preserved across multiple low-energy states, providing a structural framework that links previously independent NMR and structure–activity observations.

The relevance of such ensemble-based descriptions has become increasingly recognized in biomolecular recognition and peptide science, where low-population states can contribute disproportionately to binding and biological activity [64]. In this context, the present results move beyond a single-structure interpretation and provide a quantitative ensemble-based description of a peptide whose structural characterization has historically relied on a limited number of experimentally derived conformers and ensemble-averaged observables [57].

From a functional perspective, these findings are consistent with a conformational selection scenario [64] in which receptor binding may preferentially stabilize a subset of pre-existing low-population states rather than inducing a single ordered structure. Alternatively, an induced-fit component cannot be excluded, given the structural adaptability observed across the ensemble. In both cases, the observed conformational heterogeneity indicates that multiple structurally related states, rather than a single uniquely defined recognition conformation, may be compatible with receptor engagement, although this remains a hypothesis in the absence of explicit receptor-bound structural data. This interpretation is compatible with earlier receptor-binding studies on YIGSR-related ligands, in which recognition was proposed to depend on the spatial presentation of tyrosyl and arginyl groups rather than solely on the linear sequence [37]. In the present simulations, compact bent states may contribute to such presentation by reducing the conformational separation between the central aromatic/hydrophobic region and the C-terminal Arg-containing segment.

Recent biological studies further support the continued relevance of YIGSR-containing laminin-derived signals beyond the original anti-metastatic context. For example, YIGSR has been shown to exert concentration-dependent effects on macrophage phenotype in engineered extracellular-matrix environments, indicating that the biological output of this motif depends on presentation context as well as sequence identity [26]. Although these data do not provide structural restraints for CDPGYIGSR, they reinforce the importance of understanding how flexible YIGSR-containing motifs are presented to cellular receptors.

Several limitations of the present study should be acknowledged. First, the simulations were performed in the absence of an explicit receptor environment, and therefore the identified conformational states cannot be directly interpreted as receptor-bound conformations. Second, the receptor system historically referred to as 37/67LR remains incompletely resolved at the molecular level, making receptor-bound modeling premature in the absence of experimental binding restraints. Third, the observed conformational preferences may depend on the employed force field, water model, and terminal charge/protonation scheme. Although the available NMR data were acquired under mildly acidic conditions, Asp2 is expected to remain predominantly deprotonated under both experimental and simulated conditions because its model pKa is close to 4.0, whereas the peptide C-terminus is amidated and therefore not titratable. Consequently, protonation-state differences are unlikely to alter the main conclusions regarding the recurrent backbone motifs identified in the conformational ensemble, although they may influence the relative population of electrostatically stabilized conformations involving the D2–R9 interaction. Although GROMOS96 has been used in simulations of flexible peptides, modern force fields optimized for folded and disordered proteins may shift the relative populations of compact and extended states. Therefore, the strongest conclusions of this work concern recurrent structural motifs, ensemble heterogeneity, sampling-stable conformational features, and qualitative agreement with NMR-derived observables, rather than exact free-energy rankings. Future studies should test the robustness of these motifs using independent force fields, explicit receptor models, and experimental validation through NMR or biophysical binding assays.

Overall, this study provides a peptide-centered structural framework for interpreting CDPGYIGSR function. The central conclusion is not that a single bioactive conformation has been identified, but that the YIGSR-containing region samples a family of compact, partially preorganized states compatible with NMR and structure–activity observations. These states represent plausible starting points for future restrained docking or receptor-bound simulations, but their biological relevance will require direct validation in explicit receptor or membrane-associated environments.

## 5. Conclusions

This study characterizes the conformational ensemble of the laminin-derived peptide CDPGYIGSR in aqueous solution using temperature replica-exchange molecular dynamics. Within the sampled REMD ensemble, the peptide populates multiple conformational states rather than a single rigid structure. Across the ensemble, the most reproducible feature is a compact bend involving the YIGSR-containing region, supported by transient intramolecular hydrogen bonds and local solvent-exposure changes. Comparison with available NMR-derived distances indicates qualitative to semi-quantitative agreement with experimental observations, while also showing that no single conformation satisfies all restraints. These findings support a recognition-compatible ensemble model in which receptor engagement may involve selection or remodeling of pre-existing compact states, although direct receptor-bound simulations or experimental binding restraints will be required to validate this hypothesis. Because the laminin-binding receptor system historically referred to as 37/67LR/RPSA remains structurally complex, the present results should be viewed as a peptide-intrinsic foundation for future restrained docking, receptor-bound simulations, and experimental validation. More generally, this work reinforces the value of ensemble-based approaches for linking conformational heterogeneity to function in flexible bioactive peptides. The study also demonstrates how modern enhanced-sampling methodologies can provide new structural insight into well-established bioactive peptides by integrating conformational populations, interaction networks, and experimental observables within a unified ensemble description. Specifically, the quantitative characterization of multiple low-energy conformational basins illustrates how ensemble-based approaches can reveal information that is not directly accessible from individual experimental structures.

## Figures and Tables

**Table 1:** Main intramolecular hydrogen bonds observed across the entire 300 K ensemble. These interactions constitute the dominant hydrogen-bond network associated with compact bend-containing states and are reported as ensemble-averaged occupancies over the analyzed trajectory.

Interaction	Occupancy
D2 side chain–Y5 NH	0.39
D2 side chain–G4 NH	0.32
D2 side chain–I6 NH	0.31
D2 side chain–R9 NH	0.28
D2 side chain–G7 NH	0.25
D2 side chain–Y5 side chain	0.22
D2 side chain–R9 side chain	0.20
D2 side chain–S8 NH	0.15

**Table 2:** Comparison of interproton distances (Å) obtained from simulations and experimental NOE data. Differences are interpreted qualitatively because both simulated and experimental values represent ensemble-averaged observables. The absolute deviation |Δ| is reported as a descriptive indicator of contacts that are less well reproduced by the simulated ensemble.

Interaction	Experimental (Å)	Simulation (Å)	|Δ| (Å)
G4NH–Y5HN	2.77	3.1	0.33
Y5NH–I6HN	3.13	3.3	0.17
I6NH–G7HN	2.83	2.2	0.63
S8NH–R9HN	3.23	3.2	0.03
G4NH–G4αH	2.21	2.51	0.30
Y5NH–Y5αH	2.51	2.93	0.42
I6NH–I6αH	2.70	2.75	0.05
G7NH–G7αH	2.15	2.84	0.69
S8NH–S8αH	2.49	2.92	0.43
G4αH–Y5NH	2.15	2.43	0.28
Y5αH–I6NH	1.95	2.82	0.87
G7αH–S8NH	2.15	2.93	0.78
S8αH–R9NH	2.49	2.31	0.18

## Data Availability

The raw data supporting the conclusions of this article will be made available by the authors upon request.

## Abbreviations

The following abbreviations are used in this manuscript:

LamR	67 kDa Laminin Receptor
T-REMD	temperature replica-exchange molecular dynamics
PCA	principal component analysis
FES	free energy surface
RMSD	root mean square deviation
SASA	solvent-accessible surface area
MV	molecular volume
NOESY	Nuclear Overhauser effect spectroscopy
